# Supplementary figures and images for: Spatiotemporal control of mitotic exit during anaphase by an aurora B-Cdk1 crosstalk
Source: eLife. 2019 Aug 19;8:e47646. doi: 10.7554/eLife.47646 (PMC6706241; doi:10.7554/eLife.47646)

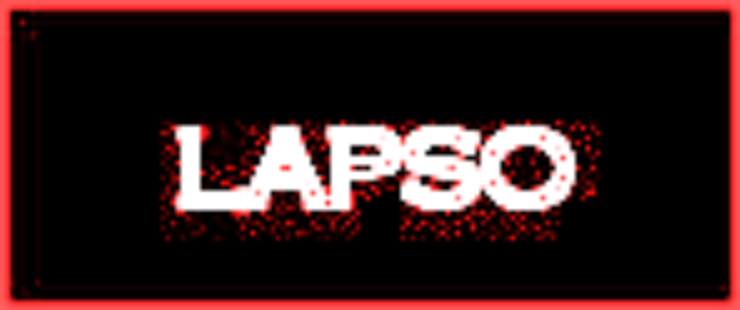

Supplement: Source code 1. [file elife-47646-code1.zip › 20190318c_LAPSO/_logoo.tif]

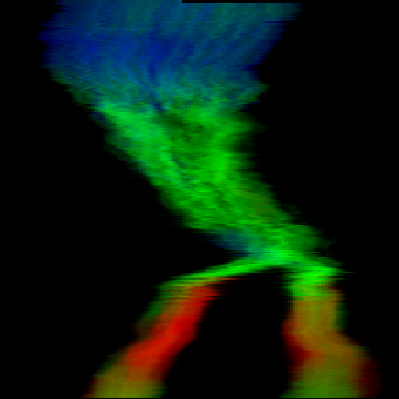

Supplement: Source code 1. [file elife-47646-code1.zip › 20190318c_LAPSO/chromokymo_example.tif]

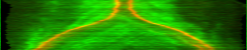

Supplement: Source code 1. [file elife-47646-code1.zip › 20190318c_LAPSO/guidedkymoExample.tif]

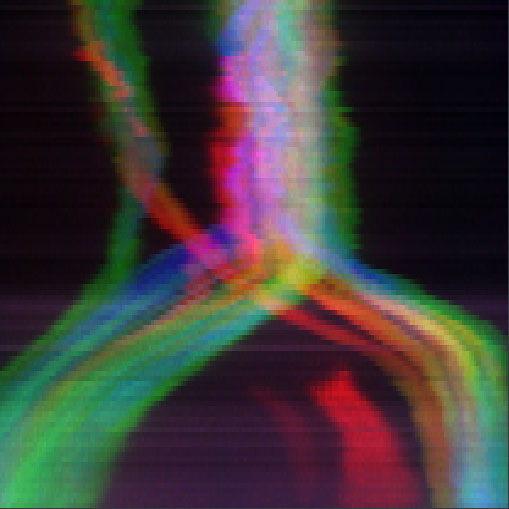

Supplement: Source code 1. [file elife-47646-code1.zip › 20190318c_LAPSO/LAPSO_frontpage.jpg]
