# Supplementary material for: Spatiotemporal control of mitotic exit during anaphase by an aurora B-Cdk1 crosstalk
Source: eLife. 2019 Aug 19;8:e47646. doi: 10.7554/eLife.47646 (PMC6706241; doi:10.7554/eLife.47646)
Supplement: Supplementary file 1. [file elife-47646-supp1.docx]

**Supplementary file 1. The CLUSTAL O(1.2.4) multiple sequence alignment.**

*NP_726244.1 – Drosophila Cyclin B1*

*EAW51306.1 – Human Cyclin B1*

*EAW77563.1 – Human Cyclin B2*

NP_726244.1 MVGTTLKMRGDENASENFKQVQLKKLTVPSMEATTK**RAAL**GDLQNRGISRPIAAKDAAQK 60

EAW51306.1 -------------------------------------MALR------------------- 4

EAW77563.1 -------------------------------------MAL-------------------- 3

**

NP_726244.1 DSKDLKL**T**DALRNAKARVDSHWKKQPLGSTNGNGNGAVPPKVNE----GGVSAFLRSNSV 116

EAW51306.1 ---------VT*RN****S****K*INAENKA------KINMAGAKRVPTAPAAT-SKPGLRP**RTAL**GDI 48

EAW77563.1 ----------LR*RP****T****V*SSDL-E------NI--------DTGVNSKVKSHVTIRRTVLEEI 38

*. . : . .:

NP_726244.1 RNRVPTK**TT**-VEPTKV**T**VKSSSSENVNEPTLKREDSNLSKK**S**LTKLRAALAKPVMGVSGI 175

EAW51306.1 GN*KV****S****E*QLQAKMPMKKEAKPSAT---------------GKVIDKKL----PKPLEKVPML 89

EAW77563.1 GNRVTTRAAQVAK--------------------------KAQNTKV----PVQ------- 61

*:* : * .*:

NP_726244.1 RREPVAVSRKEAETKKELPETKKD**S**LEVKKDATRMPLIRGNSAVTTTT-----STMPTTM 230

EAW51306.1 VPVPVSEPVPEP-----EPEPEPEPVKEE*KL****S****P*EPILVDTASPSPMETSGCAPAEEDLCQ 144

EAW77563.1 -PTKTTNVNKQL-----KPTASVKPVQMEKLAPK-----GPSPTP---EDVSMKEENLCQ 107

.: : * . . :: :* : . *

NP_726244.1 SLSSKRLAGIEDIDAND**KEN**LVLVSEYVNDIYDYLYQVELEQPIHKDHLAGQKEVSHKMR 290

EAW51306.1 AFSDVIL-AVNDVDAEDGADPNLCSEYVKDIYAYLRQLEEEQAVRPKYLLG-REVTGNMR 202

EAW77563.1 AFSDALLCKIEDIDNEDWENPQLCSDYVKDIYQYLRQLEVLQSINPHFLDG-RDINGRMR 166

::*. * ::*:* :* : * *:**:*** ** *:* * :. ..* * :::. .**

NP_726244.1 AVLIDWINEVHLQFHLAAETFQLAVAIIDRYLQVVKDTKRTYLQLVGVTALFIATKYEEL 350

EAW51306.1 AILIDWLVQVQMKFRLLQETMYMTVSIIDRFMQNNC-VPKKMLQLVGVTAMFIASKYEEM 261

EAW77563.1 AILVDWLVQVHSKFRLLQETLYMCVGIMDRFLQVQP-VSRKKLQLVGITALLLASKYEEM 225

*:*:**: :*: :*:* **: : *.*:**::* . :. *****:**:::*:****:

NP_726244.1 FPPAIGDFVFITDDTYTARQIRQMELQIFKAIDCNLSRPLPIHFL**RRYS**KAAGAEDEHHT 410

EAW51306.1 YPPEIGDFAFVTDNTYTKHQIRQMEMKILRALNFGLGRPLPLHFL**RRAS**KIGEVDVEQHT 321

EAW77563.1 FSPNIEDFVYITDNAYTSSQIREMETLILKELKFELGRPLPLHFL**RRAS**KAGEVDVEQHT 285

: * * **.::**::** ***:** *:: :. *.****:***** ** . .: *:**

NP_726244.1 MSKYFIELASVDYEMATY*RP****S****E*IAAASLFLSLHLLNGNHRAGTGFNDRHWTPTLTFYS*RY* 470

EAW51306.1 LAKYLMELTMLDYDMVHFPPSQIAAGAFCLALKILD----------NGEWTPTLQHYLSY 371

EAW77563.1 LAKYLMELTLIDYDMVHYHPSKVAAAASCLSQKVLG----------QGKWNLKQQYYTGY 335

::**::**: :**:*. : **::**.: *: ::*. : .*. . .* *

NP_726244.1 ***S****A*AHLRPITRLIAKLARD--APQAKLKAIYNKYQGSKFQKIALRTELTGALMDSIVGQSQ 528

EAW51306.1 TEESLLPVMQHLAKNVVMVNQGLTKHMTVKNKYATSKHA***KIST***LPQLNSALVQDLAKAVA 431 EAW77563.1 TENEVLEVMQHMAKNVVKVNENLTKFIAIKNKYASSKLL***KISM***IPQLNSKAVKDLASPLI 395

: : : : :** . :* :: *** ** **: :*.. :..:.

NP_726244.1 RK- 530

EAW51306.1 KV- 433

EAW77563.1 GRS 398

# box

**mutated KEN box in *Drosophila* Cyclin B1 mutated phosphorylation sites – Mathieu et al., Dev Cell, 2013 putative well conserved AuroraB phosphorylation site** *other non-conserved putative Aurora B phosphorylation sites*
